# Y-chromosomal diversity in the population of Guinea-Bissau: a multiethnic perspective

**DOI:** 10.1186/1471-2148-7-124

**Published:** 2007-07-27

**Authors:** Alexandra Rosa, Carolina Ornelas, Mark A Jobling, António Brehm, Richard Villems

**Affiliations:** 1 Department of Evolutionary Biology, Estonian Biocentre, Riia 23, 51010 Tartu, Estonia; 2 Human Genetics Laboratory, University of Madeira, Campus of Penteada, 9000-390, Funchal, Portugal; 3 Department of Genetics, University of Leicester, Leicester, LE1 7RH, UK

## Abstract

**Background:**

The geographic and ethnolinguistic differentiation of many African Y-chromosomal lineages provides an opportunity to evaluate human migration episodes and admixture processes, in a pan-continental context. The analysis of the paternal genetic structure of Equatorial West Africans carried out to date leaves their origins and relationships unclear, and raises questions about the existence of major demographic phenomena analogous to the large-scale Bantu expansions. To address this, we have analysed the variation of 31 binary and 11 microsatellite markers on the non-recombining portion of the Y chromosome in Guinea-Bissau samples of diverse ethnic affiliations, some not studied before.

**Results:**

The Guinea-Bissau Y chromosome pool is characterized by low haplogroup diversity (D = 0.470, sd 0.033), with the predominant haplogroup E3a*-M2 shared among the ethnic clusters and reaching a maximum of 82.2% in the Mandenka people. The Felupe-Djola and Papel groups exhibit the highest diversity of lineages and harbor the deep-rooting haplogroups A-M91, E2-M75 and E3*-PN2, typical of Sahel's more central and eastern areas. Their genetic distinction from other groups is statistically significant (P = 0.01) though not attributable to linguistic, geographic or religious criteria. Non sub-Saharan influences were associated with the presence of haplogroup R1b-P25 and particular lineages of E3b1-M78.

**Conclusion:**

The predominance and high diversity of haplogroup E3a*-M2 suggests a demographic expansion in the equatorial western fringe, possibly supported by a local agricultural center. The paternal pool of the Mandenka and Balanta displays evidence of a particularly marked population growth among the Guineans, possibly reflecting the demographic effects of the agriculturalist lifestyle and their putative relationship to the people that introduced early cultivation practices into West Africa. The paternal background of the Felupe-Djola and Papel ethnic groups suggests a better conserved ancestral pool deriving from East Africa, from where they have supposedly migrated in recent times. Despite the overall homogeneity in a multiethnic sample, which contrasts with their social structure, minor clusters suggest the imprints of multiple peoples at different timescales: traces of ancestral inhabitants in haplogroups A-M91 and B-M60, today typical of hunter-gatherers; North African influence in E3b1-M78 Y chromosomes, probably due to trans-Saharan contacts; and R1b-P25 lineages reflecting European admixture via the North Atlantic slave trade.

## Background

Many genetic studies of sub-Saharan Y chromosome variation have paid special attention to the large-scale Bantu expansions, and the particular pool of the "relic" Central African Pygmies and the South African Khoisan [1-7], while little is known about the events that have shaped the paternal structure of Equatorial West Africans. Although anthropological evidence is scarce, the earliest traces of West Atlantic occupation by modern humans dates back 40 ky [8,9]. Later climatic changes, when around 9 kya the Sahara was at its wettest [10], created conditions for both the massive displacement of people and the spread of agriculture, reaching previously uninhabited areas and promoting admixture with isolated populations [11-14]. Although the farming practices in Sahel could have started earlier than 6 kya [15,16], firm archaeological evidence points to the domestication of local sorghum, millet and yams ~4 kya [17]. Together with the introduction of iron-smelting techniques ~2.7 kya, agriculture led ultimately to the large-scale Bantu migrations from the Gulf of Guinea to the south of the continent [18]. From the perspective of Y chromosome genetic variation, such movements are believed to have erased much of the pre-existing diversity, replacing it with the now dominant haplogroup E3a-M2 lineages [4,19,20].

**Figure 1:**
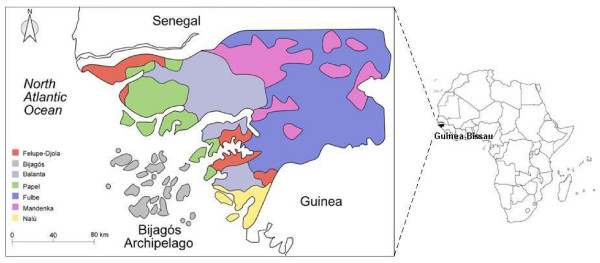
Geographic location of Guinea-Bissau and present-day settlement pattern of the ethnic groups considered in this study.

The inhabitants of the Guinea-Bissau area have certainly been under the influence of several demographic events since prehistorical times, as a result of migratory movements, trade networks and consecutive invasions. The first recorded influx of ethnically defined groups is the arrival of Fulbe people in the 8^th ^century AD, from a Central African epicenter [21]. First contact with the North African Berbers dates back to at least the 9^th ^century, and was repeated in the 11^th ^century when, pushed by the Omníades, these people came to occupy the vicinity of Senegal [22]. The economic shift in the Sahel allowed more centralized states to form (namely the "Black Kingdoms" in the period between the 8^th ^and 16^th ^centuries, [23]), linked by a trading corridor reaching from Mauritania to Niger [18]. In the following centuries pastoral Fulbe arrived again slowly but *en masse*, together with the Mandenka, and became the most prevalent people in Guinea-Bissau territory. Oral tradition also states that the Djola people – Felupe-Djola, Baiote and possibly Beafada – came from Sudan in the 15^th^-16^th ^centuries [24]. As for the Balanta, Sudanese or Bantu affinities may argue for their cultural and phenotypic aspects. Though research on the background of the Nalú is less advanced, Teixeira da Mota [25] considers them to be the autochthonous people of the region. The same author identifies Bijagós as a separated branch of Djola or relatives of Papel and Nalú. The main ethnic groups now present in Guinea-Bissau (Figure 1; see Additional file 1) were already settled in the region in the 15^th ^century, at the time of arrival of the Portuguese. With the establishment of the Atlantic slave trade the region experienced an input of Europeans, in their vast majority males, whose genetic imprint is undetermined. Many of the ethnic barriers were brought down, in particular the endogamic practices, promoting an intense cultural contact and higher levels of admixture between groups than before.

The present study intends to characterize the paternal genetic pool of Guineans, focusing on their ethnic affiliation, by the use of binary markers and microsatellites on the non-recombining region of the Y chromosome (NRY). Our sample (n = 282) extends significantly the Y-chromosomal coverage of West African populations (Senegal [5], Gambia/Senegal Wolof and Mandenka [7], Mali [2] and Dogon [7], Burkina-Faso [1,26], Ghana Ewe, Ga and Fante [7]) both in size and number of surveyed ethnic groups. The unique features of the Y chromosome system, namely its haploid and non-recombining nature and paternal inheritance, provide an opportunity to evaluate the temporal and spatial aspects of population movements, in the light of the available non-genetic evidence.

## Results and discussion

### Y chromosome haplogroup variation

**Figure 2:**
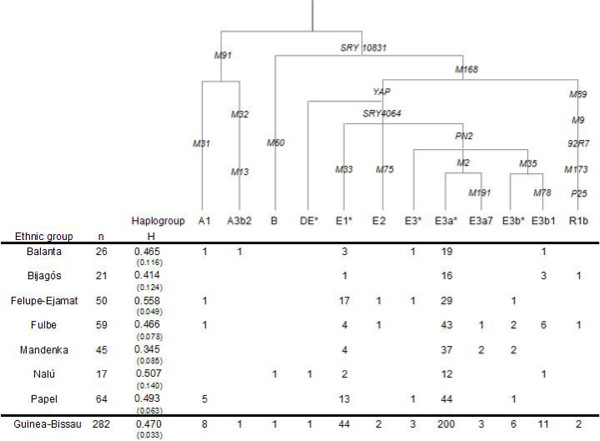
**Y chromosome haplogroup diversity in Guinea-Bissau**. Absolute numbers are shown for the total sample and ethnical clusters. Haplogroup nomenclature and defining mutations assayed in this study, shown along the branches of the phylogeny, are as proposed by the YCC [60]. The bold link indicates the root, determined by comparisons with primates [2,79].

**Figure 3:**
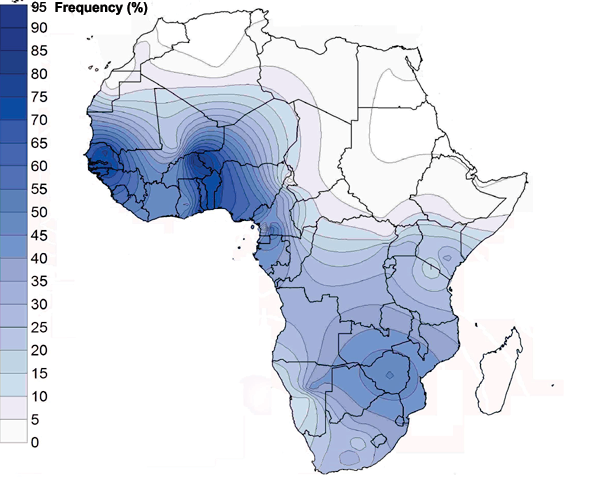
**African spatial distribution of haplogroup E3a-M2**. Frequency scale (in percentage) is shown on the left. Data according to population datasets described in Additional files 3 and 4.

The fairly homogeneous paternal structure of Guinea-Bissau (D = 0.470, sd 0.033), is not surprising given the general landscape of sub-Saharan low Y-chromosomal haplogroup diversity [2,27] and its reported east-to-west decline along a Central African corridor [28]. Responsibility for the low diversity is attributed to the highly frequent E3a*-M2 and E1*-M33 lineages (72.0% and 15.6%, respectively) that are shared among all ethnic clusters (Figure 2). In our dataset the Mandenka harbor the highest frequency (82.2%) of the E3a*-M2 paragroup, fitting the context of its closest neighbors (~80% in Senegalese [5] and Gambia/Senegal Mandinka [7]). The lack of diversity of West African Y chromosomes together with the predominance of E3a*-M2 lineages (assuming a frequency peak only equivalent to that in Central Africa; Figure 3) reinforces its link to agricultural expansion [3,4,19,20] and hint at the existence of a large local center of cultivation [14-16,18,29]. We hypothesized that the newly adopted lifestyle created conditions for major demographic growth, obscuring earlier patterns of lineages. Alternatively, a moderate farming expansion may have occurred on a background of reduced diversity, following the 5.5 kya savanna retreat [30] or the malarial epidemic episodes which were an outcome of pastoral habits [31].

The lifestyle transition in West Africa was most likely promoted by people other than the Bantu, as no relevant westwards migrations of these people are reported and none or few Bantu languages are found in the area today. In fact, the West African center may date earlier than that documented for Central Africa and may have acted as a western source of knowledge [14-16]. Based on the high frequency and microsatellite diversity of E3a*-M2 in the Mandenka and Balanta (Figure 2; see Additional file 2), we suggest that these people may have experienced a particular benefit from food production. If so, this might associate their ancestors with the people who implemented the farming habits in the Guinea-Bissau area. The Mandenka are physically and culturally descendants of the Mande, protagonists of agricultural population expansions in Niger/Mali/Burkina-Faso region [18] and rulers of the West African Black Empires, based on trade and agriculture. For the Balanta, the cultural and physical affinities with Bantu suggest a common origin at the end of the Pleistocene [24], so it may be that different peoples jointly learnt the agricultural techniques. The E3a7-M191 lineages of one Fulbe and two Mandenka individuals of Guinea-Bissau are undoubtedly representatives of a Central African lineage that followed a trajectory to the west [2,3,5,32].

Haplogroup E1*-M33, of probable local radiation (5–7% in Senegal and Burkina-Faso [2,3,5,7], 40.4% in Mali and 52.9% in Fulbe of Cameroon [1,26]), is surprisingly frequent in Felupe-Djola and Papel (34.0% and 20.3%). Both ethnic groups exhibit the highest haplogroup diversity (0.5 < D < 0.6) and the deepest-rooting phylogenetic types in our dataset – haplogroups A-M91, E2-M75 and E3*-PN2 – some with occasional occurrences in Fulbe and Balanta (Figure 2). These minor imprints may represent movements from Sahel's more central and eastern parts, seen, for example, in the typically Ethiopian/Sudanese E3*-PN2 lineages that have reached Senegambia [2,3,5]. The Djola's oral tradition claims an arrival from Sudan in the 15^th^-16^th ^centuries which is supported by their carrying the lowest fraction of E3a* in our dataset (58.0%). At the same time, the relatively short time of residence and/or the genetic isolation on cultural grounds has not contributed to a greater homogeneity among the peoples. The Papel, curiously also affiliated to the Bak-speakers, may either represent a legacy left by earlier inhabitants of the Guinean delta, survivors of an ancient pool through demographic reductions and expansions, or later arrivers who have preserved a more discrete genetic identity.

Of greater prevalence in the East quadrant of Africa and among South African Khoisan (~12% and 15%, respectively; [2,5]) the paragroup E3b*-M35 is common to Felupe-Djola and Papel (~2%) but is also found among Fulbe and Mandenka (~4%). Its presence at ~2% in Guinea-Bissau and ~5% in Senegal may also indicate loose relationships to the North, where it is widespread at rather low frequencies (2–4%, [1,26,33-35]. A similar scenario of Eastern prevalence and North African spread traces the African distribution of E3b1-M78 (~26% in Sudan and Ethiopia and 19% in NW-African Arabs), not to mention the ~7% in the Near Eastern and European people [1,5,26,33-35]. In Guinea-Bissau this haplogroup attains the highest frequency so far reported for West Africa (~4%).

The remainder of binary marker variation falls into haplogroups A, B and R, each detected at marginal frequencies (0.4–3.9%). Clades A-M91 and B-M60, the most divergent of the haplogroups of the Y chromosome tree, are associated with the earliest modern human diversification and are putative markers of the first pan-African dispersals of hunter-gatherers [2,3,7,20,36]. However, the Guinea-Bissau A-M91 lineages do not belong to the widespread A3-M32 but to the A1-M31 subcluster, with reported marginal presence in Mali (2.0% [2,7]), Gambia/Senegal Mandinka (5.1% [7]) and North African Berbers (3.1% [1,33-35]). Any association of Balanta to the Sudanese-speakers is traceable only in the A3b2-M13 and E3* Y chromosomes. The B-M60 variant observed in almost all sub-Saharan collections [28] was only found in Nalú. One other Nalú individual belongs to the rare and deep-rooting DE* paragroup described in five Nigerians [37] and thus representing a coalescent "missing link", paraphyletic to haplogroups D and E. The two Western European R1b-P25 lineages in Fulbe and Bijagós are best explained by recent European influence, at the time of the slave trade. A partial introduction through North African pastoral immigrants can not be rejected, where the 3–12% of R1b-P25 are due to the geographic proximity and the long reported contacts with Europe and Middle-East [33]. The European source seems nevertheless more likely: firstly, Y chromosome signatures of European presence have a reported great expression in the nearby Cape Verdians [38] and secondly, highly frequent North African haplogroups that would have been equally carried by the migrants (e.g. E3b2-M81) are absent in Guineans. The M173 and P25 derived states in both our samples rule out a relationship to the R1*-M173 lineage previously found in Cameroon, Oman, Egypt and Rwanda, and adduced to support the "Back-to-Africa" theory [3,28].

Pairwise F_ST _analysis of haplogroup frequencies reveals the Felupe-Djola as the only group statistically significantly different from others, namely Bijagós (F_ST _= 0.095, P = 0.027), Fulbe (F_ST _= 0.081, P = 0.004) and Mandenka (F_ST _= 0.107, P = 0.004). The exact test of population differentiation reveals similar information, further distinguishing Papel from Bijagós (P = 0.01), Fulbe (P = 0.003) and Mandenka (P = 0.04). These results are in agreement with principal components analysis (PCA; see below) and the interpretation of the greater distinctiveness of the paternal pool of Felupe-Djola and Papel among other Guineans.

### PCA and AMOVA analysis

**Figure 4:**
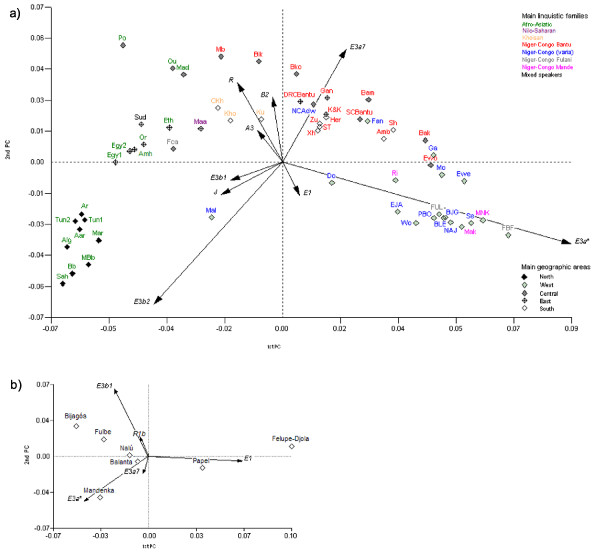
**Principal Component Analysis for a) several African populations and b) Guinea-Bissau ethnic clusters, based on haplogroup frequencies**. **a) **The 1^st ^PC captures 42.6% of the variance and 16.9% are under the responsibility of the 2^nd ^PC. For details on populational datasets see Additional file 2. The codes in italic refer to the following populations: Morocco Arabs: *Ar *[1,34], *Mar *[33]; Morocco Berbers: *Bb *[33], *MBb *[34]; Algeria: *Alg *[80], *Aar*-Algerian Arabs [35]; Tunisia-*Tun1 *[35], *Tun2 *[7]; West Sahara: *Sah*-Saharawis [33]; Egypt: *Egy1 *[35], *Egy2 *[7]; Sudan: *Sud *[2]; Ethiopia: *Eth *[2], *Or*-Oromo, *Amh*-Amhara [5,7]; Kenya: *K&K*-Kikiu & Kamba, *Maa*-Maasai [7]; Uganda: *Gan*-Ganda [7]; North Cameroon: *Po*-Podokwo, *Mad*-Mandara [7], *Ou*-Ouldeme, Daba [1,7,26], *NCAdaw*-Fali, Tali [1,26], *Fca*-Fulbe [1,26]; South Cameroon: *SCBantu*-Bassa, Ngoumba [7], *Bak*-Bakaka, [1,7], *Bam*-Bamileke [1,26], *Ewo*-Ewondo [1,26], *Bko*-Bakola Pygmies [7]; CAR: *Bik*-Biaka Pygmies [2,7]; DRC: *DRCBantu*-Nande, Herna [7]; *Mb*-Mbuti Pygmies [2,7]; Guinea-Bissau: *EJA*-Felupe-Djola, *BJG*-Bijagós, *BLE*- Balanta, *PBO*-Papel, *FUL*-Fulbe, *MNK*-Mandenka, *NAJ*-Nalú (Present study); Burkina Faso: *Mo*-Mossi [1,26], *Ri*-Rimaibe [1,26], *FBF*-Fulbe [1,26]; Gambia/Senegal: *Wo*-Wolof [7], *Mak*-Mandinka [7]; Mali: *Mal *[2], *Do*-Dogon [7]; Ghana: *Ewe, Ga, Fan*-Fante [7]; Senegal: *Se *[5]; Namibia: *Her*-Herero, *Amb*-Ambo [7], *Ku*-!Kung, Sekele [1,7,26], *CKh*-Tsumkwe San, Dama, Nama [7]; South Africa: *ST*-Sotho-Tswana, *Zu*-Zulu, *Xh*-Xhosa, *Sh*-Shona [7], *Kho*-Khoisan [2]. **b) **The PCA captures 87.0% of the variance with 74.0% and 13.0% attributed to the 1^st ^and 2^nd ^PC, respectively. The 1^st ^PC reflects an axial proportion of E3a* vs. E1* where Papel and Felupe-Djola retain the higher proportions of the later. E3a* is again a main influence in the 2^nd^axis against that of R1b and E3b1, placing Mandenka apart from Bijagós and Fulbe.

A PCA of Guinean and other African populations Y chromosome haplogroup frequencies is depicted in Figure 4a [see Additional files 3 and 4 for population details]. The 1^st ^PC clearly separates the Afro-Asiatic speakers from other linguistic families, independently of their geographic location. Consistent with geographical grouping, North and West Africans cluster in independent and tighter groups. The coordinates of North Africans are attributable to haplogroups E3b2-M81 and J-12f2 while West Africans' Y chromosomes cluster largely due to E3a*-M2, and E1-M33 to a lesser extent. Central and South African people are more dispersed in the plot, many lying closer to the Eastern populations (due to the presence of R-M207, A3-M32 and B2-M182 lineages) while others lie closer to the Western cluster. A linguistic correlation is hypothesized to underlie the genetic proximity of Bantu-speakers occupying different quadrants of the continent, driven by the E3a7-M191. Guinea-Bissau groups are included in the western cluster of populations, in close vicinity to Gambia/Senegal Wolof and Mandinka [7] and Senegalese [5] with which they share numerous population groups. It is noteworthy that the Guinea-Bissau Fulbe show a distinct pool from other Fulbe people, namely the ones in Burkina-Faso and Cameroon, and are integrated within the Guinea-Bissau variation. A PCA of Guinea-Bissau ethnic groups is illustrated in Figure 4b, less biased by the major influence of haplogroup E3a*-M2 and where the influence of minor clusters is emphasized. The Felupe-Djola and Papel have distinctive positions, largely a result of the high frequency of haplogroup E1-M33. The Bijagós, inhabitants of the archipelago, are placed apart in closer relation to the mainland Fulbe. The position of Mandenka is clearly defined by its E3a*-M2 composition.

The AMOVA yielded no statistically significant results for ethnic group distinction on any of the defined criteria, with ~97% of the variance occurring at the within-population level (P < 0.05; see Additional file 5). These results suggest that in spite of obvious sociocultural differences among groups, marked by the supposedly strict admixture barriers, their Y chromosome gene pool remains largely shared, because of common origin or common history of genetic admixture without language shift.

### Microsatellite haplotypes within haplogroups

Y-chromosomal microsatellites provide further haplotype resolution, and are of particular use when, as in this case, some haplogroups are very prevalent. The E3a*-M2 microsatellite profiles of Mandenka and Balanta are the most diverse among our data (R_ST _average gene diversity, see Additional file 2) and attest to an earlier origin or more pronounced expansion. Since the corresponding parameter in Fulbe is less diverse we consider this to signal either a genetic bottleneck or their more recent expansion and late arrival in the West. The data are consistent with the less diverse E3a-M2 profile in Central and South Africans (data not shown). Haplotypes within E3b1-M78 are supposed to represent distinct clusters of local genetic drift [39]. The rare DYS439 allele 10 of a so-called E3b1-β cluster particularly widespread among Moroccan Arabs defines a contribution to the Guinean Fulbe and Bijagós from North West Africans who have crossed the Sahara. The hypothesis of much later European contribution is valid though the remaining variability is absent (except for two R1b-P25 chromosomes) and none of the Guinean haplotypes carry the A7.1 allele with size 9, characteristic of Europe [39]. Microsatellite networks for paragroup E3a*-M2 and haplogroup E3b1-M78 are not informative due to multiple reticulations and the absence of a clear haplotype sub-structure particularly associated to ethnic groups [see Additional file 6]. Further refinement awaits the finding of new markers especially within paragroup E3a*-M2. The microsatellite profile of the DE* individual is one mutational step away from the allelic state described for Nigerians (DYS390*21, DYS388 not tested; [37], therefore suggesting a common ancestry but not elucidating the phylogenetics.

The Fulbe E3a*-M2 extended haplotypes find exact matches in Equatorial Guinea, Mozambique, Angola and Xhosa (H61, H48, H69 [40,41]; see Additional file 7) supporting their broad distribution. The Mandenka share E3a*-M2 variants with all other groups in Guinea-Bissau and do not match types outside Central-West Africa (except H67 in Mozambique), a sign of localized expansion and increased influence over their ethnic neighbors. The Felupe-Djola, Balanta and Papel each share one microsatellite haplotype (H49, H46 and H127, respectively) with Mozambique and Angola. Several E3a* eight-loci profiles matched Europeans (H29, H38, H44, H30, H152, H153 and H55), most likely descendants of incoming slaves. Three Fulbe E3b1-M78 haplotypes (H155 and H156) were found to match Spanish haplotypes [42] and samples in Central Portugal, Macedonia, Romania and Poland (YHRD database [43]). Both profiles present the A7.1 allele 12, quite frequent in Equatorial Guinea [44]. The R1b-P25 H165 has a 10-loci haplotype found in 68 worldwide populations, of which 53 are European (nine matches in Portugal, YHRD). The picture for H166-R1b is quite different since on a 7-loci basis it matches four Europeans and two individuals from the Reunion Islands, known to have a European-permeable culture.

### MtDNA haplogroup variation

Comparisons between mtDNA and Y-chromosomal diversity are hindered because of the very different mutational properties of their SNPs and Y microsatellites, and because of SNP ascertainment bias on the Y chromosome. Therefore, caution is needed when interpreting the results.

The maternal inheritance of Guineans is markedly West African, with haplogroups coalescing at distinct timeframes, from the initial occupation of the area to the later inputs of people [45]. Of relevance for comparison with the paternal counterpart are the signatures of recent expansion in haplogroups frequent in Senegambia, namely haplogroups L2a-L2c, the latter displaying an almost starlike phylogeny and being particularly frequent in the Mandenka ([45]; Tajima's D and Fu's Fs, our unpublished data). An intriguing increased frequency of L0a1 in the Balanta might parallel A1-M31 and A3b2-M13 Y chromosomes in representing East African traces. Although the founder L0a1 haplotype is shared in an east-to-west corridor, the emerging lineages are exclusive of Guineans, indicating a rapid spread and local expansion after arrival. These may therefore reflect the arrival of their ancestors in the Holocene (at about 7 kya, [45]). Moreover, the exact matches found between Balanta and North Africans in haplogroups L2a, L2b and L3b may represent evidence for their contact and long residence in the territory. L3e4 lineages, thought to signal the western expansion of food-production and iron-smelting, show a moderate frequency of 8% in the Balanta. The absence of mtDNA Bantu-markers [46-49] suggests either that Bantu people contributed very little to the maternal gene pool of Guineans, or that they had a different pool from that associated with the southwards migrations [45].

The widespread L3e2b is mainly a Felupe-Djola and Papel cluster with probable links to their homeland mirrored in exact matches with East and Central African haplotypes. Lineages within L3h, coalescing at the late Pleistocene/early Holocene in Guineans [45], exhibit one of the highest found frequencies among the Felupe-Djola (8%). Their increased frequency of West African mtDNA haplogroups L2b and L3d and Y chromosome E1*-M33 could be due to amplification in small founder groups, as these are absent in East Africa.

The mtDNA haplotypes in Guinean Fulbe exhibit a wide range of matches supported by their wide distribution and massive movements in recent history (e.g. [21]). The high frequency of L1b is otherwise a constant in the Fulbe "world" [50]. Conversely to what is seen on the paternal side, this is the only group that retains statistically significant differences in mtDNA lineages from its ethnic neighbors. As for the Y chromosome, the mtDNA pool of Bijagós shows higher affinity to that of Fulbe, making less likely any connections to the Djola, Papel or Nalú [25].

The North African mtDNA haplogroups demonstrates partial diffusion to Sahel, namely U6 found in Fulbe and Mandenka and M1b present in Guinea-Bissau Atlantic Bak-speakers ([51,52]; previously referred to as M1 in [45]). The U5b1b lineages in Fulbe and Papel are representatives of a link between the Scandinavian Saami and the North African Berbers, emphasizing the great importance of post-glacial expansions [53]. These lineages have most likely crossed the strait of Gibraltar and developed into local clusters, one of which is in West Africa. They do not seem to result from recent gene flow given that the North African Euroasiatic haplogroups H, J and T are absent in our sample.

## Conclusion

The analysis of our data provides further evidence for the homogeneity of the Y chromosome gene pool of sub-Saharan West Africans, due to the high frequency of haplogroup E3a-M2. Its frequency and diversity in West Africa are among the highest found, suggesting an early local origin and expansion in the last 20–30 ky. Hypothesizing on the existence of an important local agricultural centre, this could have supported a demographic expansion, on an E3a-M2 background, that almost erased the pre-existing Y chromosome diversity. Its pattern of diversity within Mandenka and Balanta hints at a more marked populational growth, these people possibly related to the local diffusion of agricultural expertise. The Papel and Felupe-Djola people retain traces of their East African relatives, to which the short timescale of residence in Guinea-Bissau and higher isolation from major influences have contributed. In the near absence of archaeological data, the signatures of North, Central and East Africans, traceable in less frequent extant paternal haplogroups, fit well with the linguistic and historical evidence regarding the origin and admixture processes of particular ethnic groups. Minor influences of North and East Africa, in particular, are corroborated by mtDNA data.

## Methods

### Sampling procedure

A total of 282 Guinea-Bissau unrelated healthy males were analyzed in this survey for the Y chromosome biallelic markers. The sample constitutes a subset of that typed for mtDNA [45] and therefore follows similar selection criteria and DNA extraction procedures. The present data are published as a Cape Verde source population [38] but here samples are described by ethnic affiliation. In the aforementioned article the authors were alerted to slight inconsistencies in Figure 2, which are corrected in the present work (see Figure 2), such as the missing 44 haplogroup E1*-M33 individuals. Note that discrepancies were not due to sample mistyping but to typographical errors in the original table.

In order to have a manageable number of units with reasonable sample size, many of the Guinean ethnic groups were clustered: Felupe-Djola includes the homonymous group, Baiote, Cassanga and Beafada; Papel includes Papel, Manjaco and Mancanha; Fulbe clusters Fulbe, Futa-Fulbe, Fulbe-Preto and Fulbe-Forro; Mandenka joins Mandenka, Mansonca, and Sussu; Balanta, Bijagós and Nalú were considered independently. The clustering is not without controversy, but follows pertinent information related either to history, anthropology or linguistics [24,54-59].

### Typing of Y chromosome Binary and Microsatellite Polymorphisms

The hierarchical selection of the following 31 Y chromosome binary markers according to the Y Chromosome Consortium phylogeny [60,61] allowed the inclusion of each Y chromosome into specific haplogroups: YAP [62], 92R7 [63], SRY4064, SRY10831 [64], P25 [65], PN2 [62], M2, M9, M10, M13, M14, M31, M32, M33, M35, M44, M60, M75, M78, M81, M89, M91, M116, M123, M130, M155, M168, M173, M174 and M191 [2,20]. The typing details of restriction fragment length polymorphisms (RFLPs) and direct sequencing analysis are available from the authors. The Wisconsin Package Version 10.0 [66] was employed to align DNA sequences. The nomenclature and phylogenetic relationship of lineages followed the guidelines proposed by the YCC [60], referred to in the text by the (sub)haplogroup and the terminal mutation.

The microsatellite variation, previously determined for a subset of 215 individuals [67] and newly typed for five samples, was associated to the haplogroups. Typing methodology of microsatellites DYS19, DYS389I, DYS389II, DYS390, DYS391, DYS392, DYS393, DYS385, DYS437, DYS438 and DYS439 is published elsewhere [67]. An additional GATA microsatellite A7.1 (DYS460 [68]) was tested for E3b1-M35 chromosomes.

### Data analysis

A graphical representation of the haplogroup phylogeny and distribution among ethnic clusters was built in netViz 6.5 [69]. Arlequin program ver. 2.000 [70] was used for the summary statistics on both haplogroup and microsatellite haplotype frequencies for each population unit: diversity indexes [71]; F_ST _and R_ST _calculation; exact test of population differentiation (DYS385 omitted from the analysis) [72]; AMOVA tests [73] with hierarchical clustering of the ethnic groups on geographical, linguistic and religious criteria. PCAs were performed with the software MSVP Version 3.13 m [74] for haplogroup frequencies of our data and a wide selection of African populations (units as in Figure 4; see Additional files 3 and 4), to generate a more complete picture of the African Y-haplogroup variation and the phylogeographic relationships.

Haplotype networks of microsatellite data were drawn using the Network 4.1.1.2 program [75]. Information on seven microsatellites (DYS19, DYS389I, DYS389II, DYS390, DYS391, DYS392 and DYS393) was sequentially submitted to reduced-median and median joining algorithms [76,77]. Singletons were excluded from the analysis and the threshold level of 2 was set, with weighted STR loci [78]. The YHRD database and published sources were consulted for exact matches of eight and ten microsatellites (minimal and extended haplotypes, respectively).

## Abbreviations

kya, (kilo) thousand years ago; mtDNA, mitochondrial DNA; nps, nucleotide positions; PCA, Principal Components Analysis; AMOVA, Analysis of Molecular Variance.

## Authors' contributions

AR conceived the study design and together with CO carried out the molecular genetic typing. AR performed the statistical analysis and interpreted the data to draft the manuscript. MJ, AB and RV have been involved in drafting and revising the manuscript, whose final version was read and approved by all.

## Supplementary Material

Additional file 1 **Population data on the surveyed ethnic groups of Guinea-Bissau**. The table provides information on the linguistic and religious affiliations of the Guinea-Bissau ethnic groups. Click here for file

Additional file 2 **Diversity indices and TMRCA estimates**. The table gives diversity indices for Guinea-Bissau Y chromosome haplogroups: a) coalescence time estimates for haplogroups and b) molecular diversity index (R_ST_) and TMRCA for haplogroup E3a*-M2, by ethnic group. Click here for file

Additional file 3 **Comparative African data**. The table summarizes previously published Y chromosome datasets on African populations, here considered for comparative purposes. Click here for file

Additional file 4 **Geographical distribution of African samples**. The figure displays the geographical distribution of the African Y chromosome samples considered for comparative purposes [see Additional file 3]. Click here for file

Additional file 5 **Analysis of Molecular Variance (AMOVA) in Guinea-Bissau**. The table summarizes the results of an AMOVA analysis (1023 permutations) for the Y chromosome variation among Guinean ethnic groups, clustered according to geographical, linguistic and religious criteria. Click here for file

Additional file 6 **Microsatellite haplotype networks**. The networks describe the variability of 7 microsatellite loci in Y chromosome haplogroups, among ethnic groups. a) haplogroup E3a*-M2 (N = 75, singletons excluded); b) haplogroup E3b1-M78 (N = 11), "*" denoting the E3b1-β haplotypes. Node sizes are proportional to the number of individuals. Click here for file

Additional file 7 **Haplotypes in Guinean samples**. List of the Y chromosome SNP-defined haplogroups and corresponding microsatellite haplotypes found in the Guinean sample set, by ethnic group. Click here for file
