# Supplementary material for: Y-chromosomal diversity in the population of Guinea-Bissau: a multiethnic perspective
Source: BMC Evol Biol. 2007 Jul 27;7:124. doi: 10.1186/1471-2148-7-124 (PMC1976131; doi:10.1186/1471-2148-7-124)
Supplement: Additional file 7 — Haplotypes in Guinean samples. List of the Y chromosome SNP-defined haplogroups and corresponding microsatellite haplotypes found in the Guinean sample set, by ethnic group. [file 1471-2148-7-124-S7.pdf]

Haplotype	Haplogroup	Y-STR marker												Ethnic group						
		DYS19	DYS389I	DYS389II	DYS390	DYS391	DYS392	DYS393	DYS437	DYS438	DYS439	DYS385	DYS460	Felupe-Djola	Bijagós	Balanta	Papel	Fulbe	Mandenka	Nalú
H1	A1	14	12	30	23	10	11	14	14	8	12	15,16				1				
H2	A1	14	13	30	23	11	11	14	14	8	13	16,16		1						
H3	A1	14	13	31	22	11	11	14	14	8	12	17,17					1			
H4	A1	14	13	31	22	11	11	14	14	8	14	17,19					1			
H5	A1	15	13	32	23	11	11	13	14	8	12	17,17					1			
H6	A1	15	13	32	24	11	11	14	14	8	12	17,17					1			
H7	A1	15	14	31	23	11	11	14	14	8	12	15,17					1			
H8	B	15	13	29	24	10	14	12	14	10	12	14,15								1
H9	DE	13	12	28	21	11	11	13	14	8	11	15,16								1
H10	E1*	15	12	29	22	11	10	13	17	10	11	15,16					1			
H11	E1*	15	12	29	22	11	11	13	17	10	11	15,15				1				
H12	E1*	15	12	29	22	11	11	13	17	10	12	14,14					1			
H13	E1*	15	12	30	21	10	10	13	17	10	13	14,15						1		
H14	E1*	15	12	30	21	10	11	13	17	10	12	15,16			1					
H15	E1*	15	13	29	22	11	11	14	17	10	12	14,14		1						
H16	E1*	15	13	31	24	10	10	14	17	10	12	16,17				1				
H17	E1*	15	14	29	22	10	10	14	17	10	12	14,17		1						
H18	E1*	15	14	31	22	10	11	14	17	10	13	14,16						1		
H19	E1*	16	12	29	22	10	11	13	17	8	11	15,15		1						
H20	E1*	16	12	30	22	9	11	14	16	10	13	16,16							1	
H21	E1*	16	14	29	23	11	10	13	17	10	12	15,16		1						
H22	E2	14	12	28	24	10	11	13	13	11	11	19,19						1		
H23	E3*	13	13	30	22	10	11	14	14	10	12	14,17					1			
H24	E3*	13	14	31	21	10	11	14	15	10	11	16,17				1				
H25	E3*	14	14	32	21	10	10	14	14	10	11	17,17		1						
H26	E3a	13	13	30	21	10	11	14	14	11	14	15,16							1	
H27	E3a	13	13	31	23	10	11	13	14	10	11	16,17								1
H28	E3a	13	13	31	24	10	11	14	14	10	12	16,17							1	
H29	E3a	14	13	30	21	10	11	14	15	11	13	18,18			1					
H30	E3a	14	13	30	21	10	13	14	14	11	11	16,19						1		
H31	E3a	15	12	29	21	10	11	13	13	11	12	15,16					1			
H32	E3a	15	12	29	22	10	11	13	17	10	12	14,15		1						
H33	E3a	15	12	29	22	10	11	13	17	10	13	13,17			1					
H34	E3a	15	12	29	22	10	11	13	17	10	13	15,17				1				
H35	E3a	15	12	29	22	11	10	13	16	8	11	15,15							1	
H36	E3a	15	12	29	22	11	11	13	17	10	12	14,14							1	
H37	E3a	15	12	30	21	10	11	14	13	11	11	15,16					1			
H38	E3a	15	12	30	21	10	11	14	13	11	12	15,16							1	
H39	E3a	15	12	30	21	10	11	14	13	11	13	15,16					1			
H40	E3a	15	12	30	21	10	11	14	13	11	13	16,16						1		

Haplotype	Haplogroup	Y-STR marker												Ethnic group						
		DYS19	DYS389I	DYS389II	DYS390	DYS391	DYS392	DYS393	DYS437	DYS438	DYS439	DYS385	DYS460	Felupe-Djola	Bijagós	Balanta	Papel	Fulbe	Mandenka	Nalú
H41	E3a	15	12	31	21	10	11	13	14	11	11	15,18				1				
H42	E3a	15	13	29	21	10	11	14	14	11	12	16,17					1			
H43	E3a	15	13	29	21	10	11	14	14	11	12	16,18					1			
H44	E3a	15	13	29	22	10	11	14	14	11	11	16,17		1						
H45	E3a	15	13	30	21	10	10	14	14	11	11	16,16		1						
H46	E3a	15	13	30	21	10	11	13	14	11	11	16,17				1				
H47	E3a	15	13	30	21	10	11	13	14	11	12	15,16						1		
H48	E3a	15	13	30	21	10	11	13	14	11	12	16,16						1		
H49	E3a	15	13	30	21	10	11	13	17	10	11	14,15		1						
H50	E3a	15	13	30	21	10	11	14	14	10	12	14,14					1			
H51	E3a	15	13	30	21	10	11	14	14	11	11	16,17						1		
H52	E3a	15	13	30	21	10	11	14	14	11	14	16,17							1	
H53	E3a	15	13	30	21	10	11	14	14	12	12	17,17						1		
H54	E3a	15	13	30	21	10	11	14	15	11	12	17,18			1					
H55	E3a	15	13	30	21	10	11	15	14	11	12	15,16							1	
H56	E3a	15	13	30	21	11	11	13	14	11	11	16,19						1		
H57	E3a	15	13	30	21	11	11	14	14	11	11	16,17					1			
H58	E3a	15	13	30	22	10	11	14	14	10	11	13,15							1	
H59	E3a	15	13	30	22	10	11	14	15	11	12	15,16							1	
H60	E3a	15	13	31	21	10	11	13	14	11	12	17,18						1		
H61	E3a	15	13	31	21	10	11	13	14	11	13	16,18				1				
H62	E3a	15	13	31	21	10	11	14	14	10	13	15,16				1				
H63	E3a	15	13	31	21	10	11	14	14	11	11	16,16						1		
H64	E3a	15	13	31	21	10	11	14	14	11	11	16,17						1		
H65	E3a	15	13	31	21	10	11	14	14	11	12	15,16						1	1	
H66	E3a	15	13	31	21	10	11	14	14	11	12	16,16				1				
H67	E3a	15	13	31	21	10	11	14	14	11	12	17,18							1	
H68	E3a	15	13	31	21	10	11	15	14	11	12	15,15								1
H69	E3a	15	13	31	21	11	11	13	14	11	11	16,18						1		
H70	E3a	15	13	31	22	11	11	14	14	11	12	18,18								1
H71	E3a	15	13	32	21	10	11	13	14	11	11	16,16								1
H72	E3a	15	13	32	21	10	11	13	14	11	12	17,18						1		
H73	E3a	15	13	32	21	10	11	14	14	10	13	15,16				1				
H74	E3a	15	14	30	21	10	11	14	14	11	13	16,18					1			
H75	E3a	15	14	31	21	10	11	14	14	11	12	14,14					2			
H76	E3a	15	14	31	21	10	11	14	14	11	12	16,17						1		
H77	E3a	15	14	31	21	11	11	13	14	11	12	16,18							1	
H78	E3a	15	14	31	22	10	11	13	14	11	12	15,16								1
H79	E3a	15	14	31	22	10	11	13	17	10	13	14,17							1	
H80	E3a	15	14	31	22	10	11	13	17	11	13	14,17					1			

Haplotype	Haplogroup	Y-STR marker												Ethnic group						
		DYS19	DYS389I	DYS389II	DYS390	DYS391	DYS392	DYS393	DYS437	DYS438	DYS439	DYS385	DYS460	Felupe-Djola	Bijagós	Balanta	Papel	Fulbe	Mandenka	Nalú
H81	E3a	15	14	32	21	10	11	13	14	11	11	16,17			1					
H82	E3a	15	14	32	21	10	11	13	14	11	12	16,17			1					
H83	E3a	15	14	32	21	10	11	14	14	11	12	16,16			1					
H84	E3a	16	11	28	21	10	11	13	14	11	12	16,17						1		
H85	E3a	16	12	28	21	10	11	14	14	11	12	16,16					1			
H86	E3a	16	12	30	21	10	11	14	14	11	13	15,16							1	
H87	E3a	16	12	30	21	10	11	14	14	11	13	16,16							1	
H88	E3a	16	12	30	21	11	11	14	14	11	12	17,17					1			
H89	E3a	16	13	29	21	10	11	14	14	11	11	15,18							1	
H90	E3a	16	13	29	21	10	11	14	14	11	11	16,18		1						
H91	E3a	16	13	29	21	10	11	14	14	11	12	15,18						1		
H92	E3a	16	13	29	21	10	11	14	14	11	12	16,16				1				
H93	E3a	16	13	30	21	10	11	14	14	12	12	16,16							1	
H94	E3a	16	13	30	21	11	11	14	14	11	11	17,17		1						
H95	E3a	16	13	30	21	11	11	14	14	11	12	17,18						1		
H96	E3a	16	13	30	22	10	11	14	14	12	12	16,16		1						
H97	E3a	16	13	30	22	10	11	14	15	11	11	15,17							1	
H98	E3a	16	13	31	21	10	11	14	14	10	12	16,16					1			
H99	E3a	16	13	31	21	10	11	14	14	11	10	16,16		1						
H100	E3a	16	13	31	21	10	11	14	14	12	11	16,16			1					
H101	E3a	16	13	31	21	10	11	15	14	10	12	16,17		1						
H102	E3a	16	13	31	22	10	11	14	14	11	12	14,17								1
H103	E3a	16	13	32	21	10	11	13	14	11	12	17,17		1						
H104	E3a	16	13	32	21	10	11	14	14	11	10	16,16							1	
H105	E3a	16	13	32	21	11	11	13	14	11	11	17,18				1				
H106	E3a	16	13	32	21	12	11	14	14	11	13	15,17						1		
H107	E3a	16	14	31	21	10	11	13	14	11	12	15,17						1		
H108	E3a	16	14	31	21	10	11	14	14	10	12	16,18								1
H109	E3a	16	14	31	21	10	11	14	14	11	11	17,18		1						
H110	E3a	16	14	31	21	10	11	14	14	11	12	16,16			1					
H111	E3a	16	14	31	21	10	12	14	15	12	11	18,19			1					
H112	E3a	16	14	31	22	10	11	13	14	11	13	16,16							1	
H113	E3a	16	14	31	22	10	11	13	14	11	14	16,17				1				
H114	E3a	16	14	31	22	10	11	13	15	11	12	16,17					1			
H115	E3a	16	14	31	22	10	11	14	14	11	11	15,17					1			
H116	E3a	16	14	31	22	10	11	14	15	11	11	16,18				1				
H117	E3a	16	14	32	21	10	11	13	14	11	12	15,15							1	
H118	E3a	16	14	32	21	10	11	14	14	11	11	15,17				1				
H119	E3a	16	14	32	21	10	11	14	15	11	12	17,20			1					
H120	E3a	16	14	32	21	10	11	14	15	11	13	20,21			1					

Haplotype	Haplogroup	Y-STR marker												Ethnic group						
		DYS19	DYS389I	DYS389II	DYS390	DYS391	DYS392	DYS393	DYS437	DYS438	DYS439	DYS385	DYS460	Felupe-Djola	Bijagós	Balanta	Papel	Fulbe	Mandenka	Nalú
H121	E3a	17	12	29	21	10	11	14	14	11	13	17,17		2						
H122	E3a	17	12	30	21	10	11	14	15	11	13	15,16					1			
H123	E3a	17	12	32	22	11	11	13	17	8	11	15,15				1				
H124	E3a	17	13	29	21	10	11	14	14	11	12	15,18								1
H125	E3a	17	13	30	21	10	11	13	14	11	12	17,18		1						
H126	E3a	17	13	30	21	10	11	13	14	11	13	17,18		1						
H127	E3a	17	13	30	21	10	11	14	14	11	11	17,17					2			
H128	E3a	17	13	30	21	10	11	14	15	11	11	16,17					1			
H129	E3a	17	13	30	21	11	11	13	14	11	11	17,17					1			
H130	E3a	17	13	31	20	11	11	15	14	11	11	16,16							1	
H131	E3a	17	13	31	21	10	11	13	14	11	10	15,16					1			
H132	E3a	17	13	31	21	10	11	14	14	11	10	16,16				1				
H133	E3a	17	13	31	21	10	11	14	14	11	12	17,17								1
H134	E3a	17	13	32	20	10	11	14	14	11	11	15,16		1						
H135	E3a	17	13	32	21	10	11	13	14	11	10	16,17		1						
H136	E3a	17	13	32	21	10	11	15	14	11	11	15,16		1						
H137	E3a	17	13	32	22	10	11	13	15	11	13	16,17							1	
H138	E3a	17	14	31	21	10	11	13	14	11	12	16,16		1						
H139	E3a	17	14	31	21	10	11	14	15	11	12	18,20			1					
H140	E3a	17	14	31	21	10	11	15	15	11	12	18,20			1					
H141	E3a	17	14	31	21	11	10	14	14	11	12	16,18				1				
H142	E3a	17	14	31	21	11	11	14	15	11	12	18,18			1					
H143	E3a	17	14	31	22	10	11	13	14	11	12	15,17						1		
H144	E3a	17	14	32	21	10	11	14	14	11	11	17,17						1		
H145	E3a	17	15	31	22	10	12	13	15	11	13	17,17				1				
H146	E3a7	15	12	30	21	10	11	14	13	11	12	15,16							1	
H147	E3a7	16	14	31	21	10	11	13	14	11	13	17,17						1		
H148	E3a7	17	13	30	21	10	12	14	14	11	13	18,18							1	
H149	E3b*	13	12	30	22	9	11	13	14	10	13	14,17						1		
H150	E3b*	13	13	29	24	11	11	14	14	10	13	14,16						1		
H151	E3b*	13	13	30	22	9	11	12	14	10	13	13,16					1			
H152	E3b*	13	13	30	22	9	12	12	14	10	12	16,17							1	
H153	E3b*	13	13	30	23	10	13	13	14	11	12	16,16							1	
H154	E3b*	13	13	30	24	10	10	13	14	10	13	15,16		1						
H155	E3b1	13	13	30	24	10	11	13	14	10	12	16,17	12					2		
H156	E3b1	13	13	30	24	10	11	13	14	10	12	17,17	12					1		
H157	E3b1	13	13	30	24	10	11	14	14	10	13	16,16	11					1		
H158	E3b1	13	13	30	24	10	12	13	15	10	12	15,15	11		1					
H159	E3b1	13	13	31	24	10	12	14	15	10	13	16,16	12			1				
H160	E3b1	13	13	31	23	10	11	13	14	10	11	15,16	11							1

Haplotype	Haplogroup	Y-STR marker												Ethnic group							
		DYS19	DYS389I	DYS389II	DYS390	DYS391	DYS392	DYS393	DYS437	DYS438	DYS439	DYS385	DYS460	Felupe-Djola	Bijagós	Balanta	Papel	Fulbe	Mandenka	Nalú
H161	E3b1	13	13	31	24	10	12	12	15	10	12	15,15	11		1					
H162	E3b1	13	13	31	24	11	11	13	14	10	10	15,16	10					1		
H163	E3b1	13	13	32	25	11	11	13	15	10	12	15,18	11					1		
H164	E3b1	14	13	30	23	10	11	14	14	10	10	13,14	12		1					
H165	R1b	14	13	29	24	10	13	13	15	12	12	11,14			1					
H166	R1b	15	14	31	25	10	13	13	14	12	13	13,15						1		
H167	E1*		12	29	22	10	10	14		12	11	15,16		1						
H168	E1*		12	29	22	11		13			11	15,16		1						
H169	E1*		12	30	22	11		13			12	13,16					1			
H170	E1*		13	30	22	10		13			12	13,16					1			
H171	E1*		14		22	10		13			13	15,17				1				
H172	E3a		11	30	21	10		14			11	16,17							1	
H173	E3a	16	11		21	10		13	14									1		
H174	E3a		12	29	21	10		14			12	16,19					1			
H175	E3a		12	29	21	10		14			14	16,17		1						
H176	E3a		12	29	21	11		14			12	17,18							1	
H177	E3a		12	29	21	11		14	14		13	16,17		1						
H178	E3a		12	29	22	11		13			11	15,16		1						
H179	E3a		12	29	22	11		13			12	14,14					1			
H180	E3a		12	30	21	10		14			12	16,16						1		
H181	E3a		12	30	21	10		14			13	14,17				1				
H182	E3a		12	30	21	10		14			13	15,16						1		
H183	E3a		13	28	21	10		13			12	16,17						1		
H184	E3a		13	29	21	10		13			12	16,16						1		
H185	E3a		13	29	21	10		14			12	16,18					1			
H186	E3a		13	29	21	10		14			12									1
H187	E3a		13	30	20	10		14			11	15,16						1		
H188	E3a		13	30	21	9		13			13	15,15						1		
H189	E3a		13	30	21	9		13			13	15,16						1		
H190	E3a		13	30	21	10		13			12	16,16								1
H191	E3a		13	30	21	10		14			12	14,14					1			
H192	E3a		13	30	21	10		14			12	15,16						1		
H193	E3a		13	30	21	10		14			12	17,17						1		
H194	E3a		13	30	21	10		14			12	17,18		1						
H195	E3a		13	30	21	10		14			13	16,18						1		
H196	E3a		13	30	21	10		15			11	16,19							1	
H197	E3a		13	30	21	10		15			12	15,15							1	
H198	E3a		13	30	21	10		15			12	16,16			1					
H199	E3a		13	30	21	11		12			11	17,17							1	
H200	E3a		13	30	21	11		13			11	16,17					1			

Haplotype	Haplogroup	Y-STR marker												Ethnic group						
		DYS19	DYS389I	DYS389II	DYS390	DYS391	DYS392	DYS393	DYS437	DYS438	DYS439	DYS385	DYS460	Felupe-Djola	Bijagós	Balanta	Papel	Fulbe	Mandenka	Nalú
H201	E3a		13	30	21	11		13			11	16,20						1		
H202	E3a		13	30	21	11		14			11	17,17					1			
H203	E3a		13	30	21	11		14			11	17,18		1						
H204	E3a		13	31	21	10		14			10	16,16		1						
H205	E3a		13	31	21	10		14			10	16,16		1						
H206	E3a		13	31	21	10		14			11	15,16						1		
H207	E3a		13	31	21	10		14			12	17,18						1		
H208	E3a		13	31	22	10		14	15	12	13	16,17							1	
H209	E3a		13	31	23	10		13			11	16,18								1
H210	E3a		13	32	21	11		13			12	17,17						1		
H211	E3a		14	31	21	9		13			12	15,16				1				
H212	E3a		14	31	21	10		15			11	16,16							1	
H213	E3a		14	31	22	10		13			13	15,17				1				
H214	E3a		14	32	21	10		14			10	16,16					1			
H215	E3a		14	32	21	10		14			11	16,17							1	
H216	E3a		14	32	21	10		14			12	14,14					1			
H217	E3a		14	33	21	11		14			12	15,16		1						
H218	E3a	16	14		21	10	10	14	14	8	13				1					
H219	E3a		14		21	10		15	14	12	11				1					
H220	E3b1		13		24	10		13			12	16,16			1					
